# Endobronchial Watanabe Spigots for Treatment of Pyopneumothorax due to Nontuberculous Mycobacteriosis in a Patient with Rheumatoid Arthritis: A Case Report Based on Past Experience

**DOI:** 10.1155/2018/6738435

**Published:** 2018-09-26

**Authors:** Norikazu Kawai, Takeshi Kawaguchi, Takashi Tojo, Takao Osa, Yoshifumi Yamamoto, Motoaki Yasukawa, Noriyoshi Sawabata, Shigeki Taniguchi

**Affiliations:** ^1^Department of Thoracic and Cardiovascular Surgery, Nara Medical University, Kashihara, Nara, Japan; ^2^Second Department of Internal Medicine, Nara Medical University, Kashihara, Nara, Japan

## Abstract

Nontuberculous mycobacterial lung disease sometimes causes pneumothorax and empyema, which are often intractable because of patients' background factors. Biological products used in the treatment of rheumatoid arthritis have caused the problem of an increase in infection rates as a side effect, one of which is nontuberculous mycobacteriosis (NTM). On the basis of past experience, we report the case of a patient who had a history of undergoing treatment with biological products against rheumatoid arthritis. The patient was treated for NTM-induced pyopneumothorax by endoscopic bronchial occlusion therapy using endobronchial Watanabe spigots.

## 1. Introduction

Pneumothorax and empyema are uncommon but serious complications of nontuberculous mycobacteriosis (NTM) disease [1]. Secondary pneumothorax and empyema due to NTM may require other treatment in addition to chemotherapy and thoracic drainage. Conventional surgical treatment is one of the options for this condition, although postoperative recurrence and chronicity have also been reported and there are some cases for which surgical care is expected to be extremely difficult [2]. Nonetheless, endoscopic bronchial occlusion with the endobronchial Watanabe spigot (EWS) has been reported to be useful for pneumothorax and empyema and is less invasive than surgical treatment [3–5]. We report here a successful case of bronchial occlusion therapy using the EWS to treat pyopneumothorax associated with NTM in a patient who was undergoing treatment with biological products against rheumatoid arthritis (RA), based on our past experience.

## 2. Case Presentation

**Figure 1:**
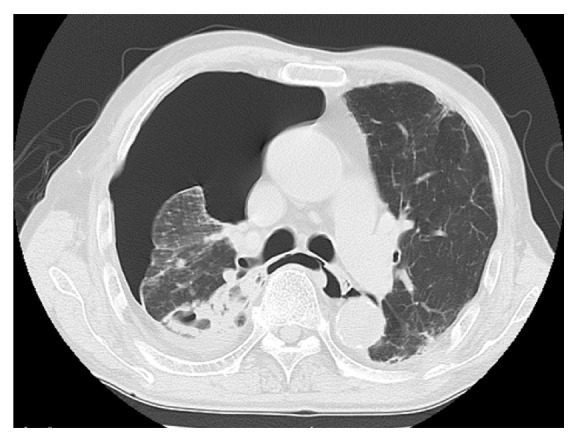
Chest computed tomography scan shows pneumothorax and pleural effusion in the right pleural cavity and a cavity lesion in the right upper lobe.

**Figure 2:**
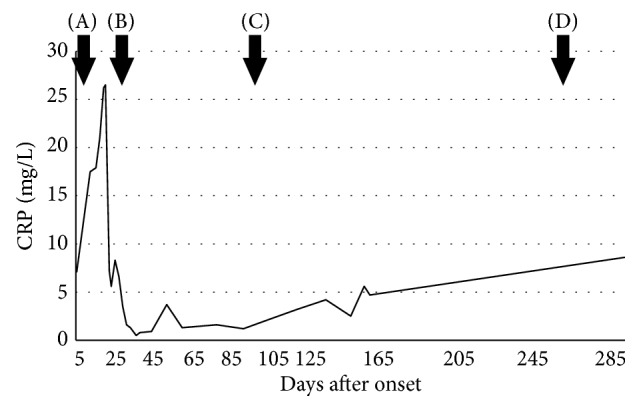
Clinical course and CRP concentration. (A) The first session of endobronchial Watanabe spigot application. (B) The second session of endobronchial Watanabe spigot application. (C) Cessation of air leakage. (D) Thoracic drain removal. CRP, C-reactive protein.

**Figure 3:**
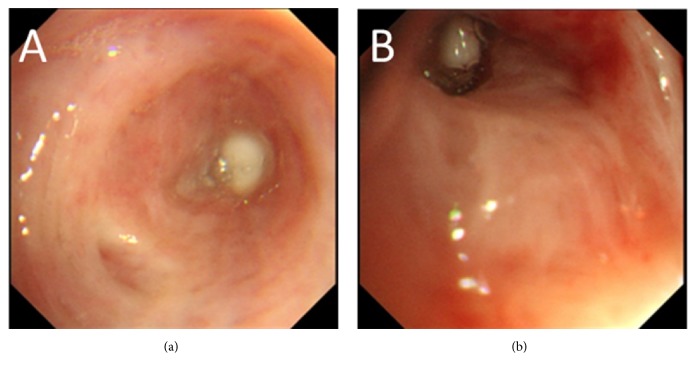
(a) Endoscopy showed a bronchial occlusion in the right B^2^ at the first session using an endobronchial Watanabe spigot. (b) Endoscopy showed a bronchial occlusion in the right B^1^b at the second session using an endobronchial Watanabe spigot.

**Figure 4:**
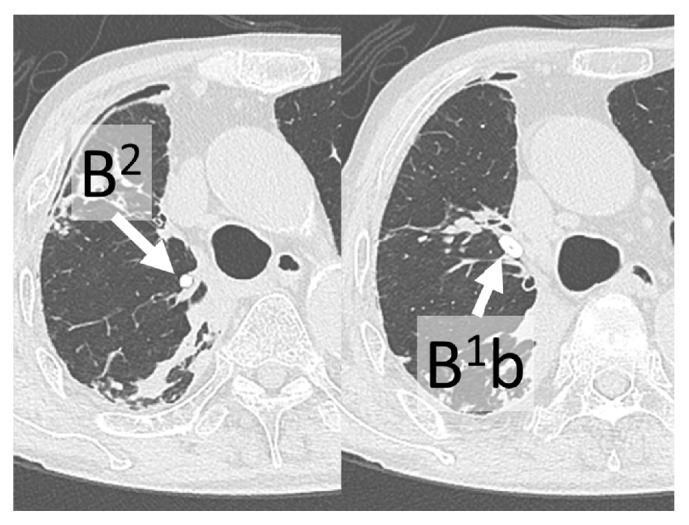
Chest computed tomography shows improvement of pneumothorax and pleural effusion in the right lung. Endobronchial Watanabe spigots were placed in B^2^ and B^1^b of the right lung.

A 74-year-old male outpatient had been undergoing treatment for RA since 2000 and began taking the biological product tocilizumab in 2014. This treatment was stopped in 2015 because of excreted* Mycobacterium intracellulare* in the sputum. He was admitted to our hospital because of breathing difficulty in May 2016. A chest computed tomography scan showed pneumothorax and pleural effusion in the right lung and a main cavity lesion in the right upper lobe (Figure 1). A laboratory culture from the sputum and pleural effusion was positive for* M. intracellulare*, and we arrived at a diagnosis of secondary pyopneumothorax caused by* M. intracellulare*. The changes in the C-reactive protein (CRP) concentration are shown in Figure 2. Despite multidrug therapy and thoracic drainage for the pyopneumothorax, air leakage persisted. We planned endoscopic bronchial occlusion therapy because surgical therapy carried a high risk for the patient (his performance status at the time was 2). This technique was performed in the right B^2^ bronchus for the first session on day 11 (Figures 3(a) and 4), but it did not completely stop the air leak. Therefore, we performed the second session in B^1^b on day 32 after onset (Figures 3(b) and 4). Following the second treatment, although major air leakage was not observed a minor air leak persisted for some time, eventually stopping on day 99. Thereafter, thoracic drainage was continued because of the prolonged purulent discharge [a laboratory culture of the pleural effusion was negative for* M. intracellulare* on day 138, whereas it had been positive for methicillin-resistant* Staphylococcus aureus* from day 87 to day 228 (the final examination day)]. The purulent discharge was then reduced, the thoracic drain was removed on day 263, and the pyopneumothorax improved. At the time of the present writing (>2 years after the procedure), the patient was undergoing follow-up on an outpatient basis.

## 3. Discussion

In the present case, bronchial occlusion using the EWS was effective for a patient with pyopneumothorax associated with NTM who had a history of undergoing treatment with biological products against RA.

In epidemiological studies on NTM in the United States, the incidence of NTM in patients with RA was 19.2 per 10^5^ patient-years. Additionally, the incidence rate of NTM increased 5-fold for patients who were under treatment with biological products [6]. With regard to NTM therapy, there is no difference in the standard care of patients with RA who are under treatment with or without biological products. However, regarding prognosis, patients with NTM and RA complications have a 5-year survival rate of 66.1%, which is lower than that of NTM alone [7]. NTM sometimes causes secondary pneumothorax and empyema, which might be refractory because of the patient's background factors. When RA is complicated, such as in our case, the general condition is usually poor, and surgical therapy is often presumed to be over-invasive.

Bronchial occlusion is useful for controlling pneumothorax and empyema complicated by NTM [3], and we also previously reported a similar case of effective use of EWS for pyopneumothorax secondary to NTM in a patient who underwent treatment with biological products against RA [4] (Table 1). Endoscopic bronchial occlusion has been practiced since a report by Rafinski in 1965 [8], and the EWS, which is made from silicone material, has been used recently for endoscopic bronchial occlusion therapy [5]. In the current case, although surgical treatment was also considered we chose endoscopic bronchial occlusion because of the patient's condition and his desire to avoid surgery. The first treatment was inadequate, but air leakage was stopped after the second treatment. However, as prolonged purulent discharge was observed, thoracic drainage was continued. On day 148, we attempted to release negative pressure of the thoracic drain to enable the patient to manage the drainage tube himself after leaving the hospital. Because collapse of the lung was mild, we changed the drainage tube to an open drain, and the patient was discharged from hospital on day 160. After this time the dead space in the thoracic cavity naturally narrowed, and the purulent discharge was reduced. Although the laboratory data (CRP concentration and pleural effusion culture results) were not negative, we removed the thoracic drain on day 263 because the CRP concentration was also influenced by the condition of the RA, the pleural effusion had been drained, and the patient's general condition was stable. Although treatment was required for a long period, the pyopneumothorax improved without loss of the patient's performance status.

As already stated, endoscopic bronchial occlusion with EWS sometimes does not completely stop air leakage, and some reports have mentioned that multiple treatments or additional treatment is required. Moreover, even if the air leak is stopped the purulent discharge may persist, meaning that drainage over a long duration may be required (Table 1). There is also concern when an NTM lesion in the lungs remains and a silicone stent is placed in the bronchus. Although other unknown problems may exist, endoscopic bronchial occlusion is much less invasive than surgical treatment and represents a useful option, especially for patients with a high risk for surgery.

In conclusion, for patients with pyopneumothorax secondary to NTM who have a history of undergoing treatment with biological products against RA, bronchial occlusion with the EWS is a potential treatment option.

## Figures and Tables

**(a):** 

	Age (year)	Sex	Location of the lung	Causative bacteris
Case 1 [4]	73	Male	Right upper lobe	*Mycobacterium intracellulare*
Case 2 (present case)	74	Male	Right upper lobe	*Mycobacterium intracellulare*

**(b):** 

	Number of EWS	Duration of air leak after EWS (day)	Drainage period after ESW (day)
Case 1 [4]	2 times to B^3^	0	110
Case 2 (present case)	2 times to B^2^ and B^1^b	67	231

EWS, endobronchial Watanabe spigots; NTM, nontuberculous mycobacteriosis; RA, rheumatoid arthritis.
